# Intravenous administration of normal saline may be misinterpreted as a change of end-expiratory lung volume when using electrical impedance tomography

**DOI:** 10.1038/s41598-019-42241-7

**Published:** 2019-04-08

**Authors:** Vladimír Sobota, Martin Müller, Karel Roubík

**Affiliations:** 1 Department of Biomedical Technology, Faculty of Biomedical Engineering, Czech Technical University in Prague, Kladno, Czech Republic; 2 Department of Physiology, Maastricht University, Maastricht, The Netherlands; 3 Department of Anaesthesiology and Intensive Care, First Faculty of Medicine, Charles University and Thomayer Hospital, Prague, Czech Republic

## Abstract

Electrical impedance tomography (EIT) is a noninvasive imaging modality that allows real-time monitoring of regional lung ventilation. The aim of the study is to investigate whether fast saline infusion causes changes in lung impedance that could affect the interpretation of EIT data. Eleven pigs were anaesthetized and mechanically ventilated. A bolus of 500 mL of normal saline was administered rapidly. Two PEEP steps were performed to allow quantification of the effect of normal saline on lung impedance. The mean change of end-expiratory lung impedance (EELI) caused by the saline bolus was equivalent to a virtual decrease of end-expiratory lung volume (EELV) by 227 (188–250) mL and decremental PEEP step of 4.40 (3.95–4.59) cmH_2_O (median and interquartile range). In contrast to the changes of PEEP, the administration of normal saline did not cause any significant differences in measured EELV, regional distribution of lung ventilation determined by EIT or in extravascular lung water and intrathoracic blood volume. In conclusion, EELI can be affected by the changes of EELV as well as by the administration of normal saline. These two phenomena can be distinguished by analysis of regional distribution of lung ventilation.

## Introduction

**Figure 1:**
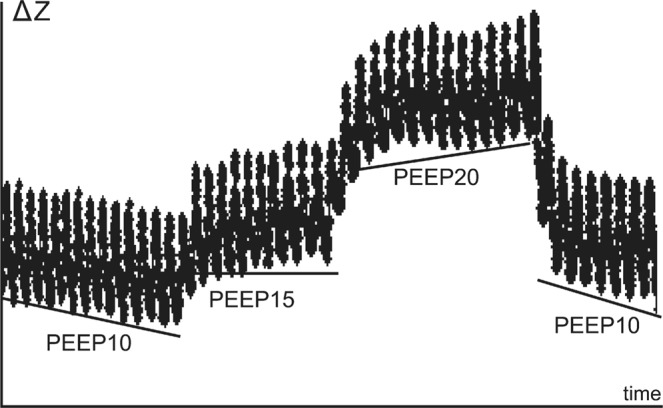
Optimization of positive end-expiratory pressure (PEEP) using electrical impedance tomography. Decreasing trend in end-expiratory lung impedance (EELI) indicates derecruitment while an increase of EELI indicates recruitment. Horizontal tracing indicates stable end-expiratory lung volume and corresponds with the optimal PEEP. Reproduced with permission^7^.

Electrical impedance tomography (EIT) is a noninvasive bedside imaging modality suitable for continuous monitoring of lung functions^1^. Dividing the acquired images into regions of interest (ROIs), EIT provides information about regional distribution of lung ventilation^2–4^. Using the global trend of end-expiratory lung impedance (EELI), changes in end-expiratory lung volume (EELV) can be estimated^5,6^. EIT is considered to be an easy-to-use technique for PEEP optimization^7–9^, as shown in Fig. 1. The decreasing trend of EELI represents a gradual alveolar derecruitment due to insufficient PEEP, while an increasing trend represents recruitment. A stable EELI value indicates the optimal PEEP level.

During our previous experiments in pigs with EIT measurements lasting several hours we observed a noticeable steady decrease of EELI values. This decrease could be falsely interpreted as a change of EELV that was not apparent. Importantly, we observed that the decreasing trend of EELI changed immediately after an interruption of intravenous fluid administration and was restored again when the fluid administration was re-established.

The effect of fluid balance on changes in electrical impedance of human body is well known and widely used in bioelectrical impedance analysis for determination of total body water^10,11^. There are studies that described the effect of fluid accumulation in peritoneum^12,13^, diuresis^14^ or intravenous fluid administration^15^ upon the changes of thoracic impedance as determined by EIT. However, to the best of our knowledge, there is no study dealing with the direct comparison of lung volume related changes in EIT with those caused by fluid administration.

The aims of the study are (1) to investigate whether the fast intravenous administration of normal saline affects EELI in healthy porcine lungs, (2) to compare these impedance changes with the impedance changes associated with PEEP alterations, (3) to investigate whether the impedance changes caused by intravenous administration of normal saline can be distinguished from the impedance changes caused by PEEP alterations.

## Materials and Methods

The prospective interventional animal study was performed in the accredited animal laboratory of the Department of Physiology, First Faculty of Medicine, Charles University in Prague, in accordance with Act No. 246/1992 Coll., on the protection of animals against cruelty that incorporates the relevant legislation of the European Community. The study was approved by the Institutional Animal Care and Use Committee of the First Faculty of Medicine, Charles University in Prague.

### Animal preparation and monitoring

Eleven crossbred (Landrace × Large White) female pigs (*Sus scrofa domestica*) 3–4 months old with an average body weight of 48 kg (43–52 kg range) were involved in the study.

The animals received total intravenous anaesthesia with muscle relaxation. Mechanical lung ventilation was conducted using an Engström Carestation (GE Healthcare, Waukesha, WI, USA) ventilator in the volume-controlled mode. The pre-set and measured ventilatory parameters are described in Table 1. Detailed information regarding the anaesthesia and animal preparation is provided in the Supplementary Information.

**Table 1:** Ventilatory parameters during the study protocol.

Parameter	Baseline values (Phase 1)	End values (Phase 6)	*p* Wilcoxon test
Expiratory tidal volume, mL	469 (426–494)	473 (424–498)	0.91
Respiratory rate*, b/min	20 (20–22)	20 (20–22)	—
Inspiratory to expiratory time ratio*	0.5	0.5	—
Fraction of inspired oxygen*, %	25 (25–30)	25 (25–30)	—
End-tidal carbon dioxide concentration, mmHg	43 (40–44)	42 (40–44)	0.12
Peripheral capillary oxygen saturation, %	98 (97–100)	98 (96–100)	0.91
Minute ventilation, L/min	9.2 (8.9–9.5)	9.3 (8.9–9.5)	0.47
Peak airway pressure, cmH_2_O	23 (21–26)	25 (24–26)	0.07
Positive end-expiratory pressure*, cmH_2_O	5	5	—
Compliance, ml/cmH_2_O	32 (31–42)	31 (30–39)	0.07

Data are presented as median and interquartile range (Q_1_–Q_3_).

*Preset parameter.

EIT system PulmoVista 500 (Dräger Medical, Lübeck, Germany) was used for data acquisition. An electrode belt of size “S” (chest circumference from 70 to 85 cm) was attached to the animal chest, cranially to the level of diaphragm at PEEP of 5 cmH_2_O. In most of the subjects, this position corresponded with the 6^th^ intercostal space. Correct placement of the electrode belt was verified by chest X-ray. The frequency of the applied alternating current was set to 110 kHz and the EIT images were acquired with a frame rate of 50 Hz.

End-expiratory lung volume (EELV) was measured by FRC INview module of the Engström Carestation ventilator. The applied change of FiO_2_ for the wash-in/wash-out measurement of EELV was 10%.

Haemodynamic monitoring was performed using Edwards LifeSciences monitor EV1000 (Edwards LifeSicences, Irvine, CA, USA). Cardiac output, extravascular lung volume (EVLW) and intrathoracic blood volume (ITBV) were measured using the technique of transpulmonary thermodilution (TPTD) with the PiCCO arterial catheter placed in *a. femoralis* and cold normal saline applied in *v. jugularis interna*. Maximum of six boluses of normal saline were applied, 10 mL each. According to the EV1000 reference manual, three values of CO that did not differ more than by 10% were used for the evaluation.

### Study protocol

**Figure 2:**
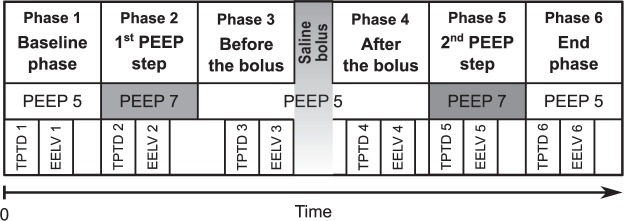
Timeline of the study protocol. TPTD1–TPTD6: individual transpulmonary thermodilution measurements; EELV1–EELV6: individual end-expiratory lung volume measurements.

After preparation, instrumentation and myorelaxation of an animal, calibration of the EIT system was performed and data acquisition was initiated. Recording of ventilatory data was initiated as well. A steady phase in a duration of approximately 30 minutes was introduced. The study protocol consisted of six phases and is described schematically in Fig. 2. Initial TPTD measurement was conducted at the beginning of the baseline phase. Incremental PEEP step from 5 to 7 cmH_2_O was performed and the second TPTD measurement followed. PEEP was decreased back from 7 to 5 cmH_2_O and TPTD was measured again. Consequently, a bolus of 500 mL of normal saline was administered using a pressure infusion bag. The duration of the administration was 6 minutes on average. Three minutes after the end of the saline administration a TPTD was measured, followed by the second incremental PEEP step from 5 to 7 cmH_2_O. TPTD was measured again and PEEP was decreased back to the initial value. The last TPTD measurement was done during the end phase when the PEEP was set back to 5 cmH_2_O. EELV measurements were performed always immediately after the measurements of TPTD.

### Data analysis and statistics

EIT data were pre-processed using EIT Data Analysis Tool (Dräger Medical, Lübeck, Germany). For each animal, a reference frame (also referred to as a baseline frame) was selected from the initial baseline phase of the experiment and was used for the reconstruction of EIT images and impedance waveforms. The data were exported to MATLAB (Mathworks Inc., Natick, MA, USA) where further processing was performed.

For each breath cycle, tidal variation (TV) image was calculated as a difference between the end-inspiratory and the previous end-expiratory EIT image^1^. Four horizontal regions of interest (ROIs) were determined in the TV images, numbered from 1 (ventral) to 4 (dorsal). Proportional ventilation was calculated for each ROI and was expressed as a percentage of the overall tidal impedance change. Mean values of proportional ventilation were calculated for each phase, considering the values from an interval of 60 seconds. Detailed description of the method for calculation of the differences in regional ventilation is provided in the Supplementary Information.

To quantify the changes in end-expiratory lung impedance caused by the administration of 500 mL of normal saline (ΔZ_bolus_), the value of ΔZ_bolus_ was expressed both as an equivalent (i.e. virtual) change of end-expiratory lung volume (ΔEELV_bolus,equiv_) and an equivalent (virtual) change of positive end-expiratory pressure (PEEP_bolus,equiv_). The values of ΔEELV_bolus,equiv_ were compared with reference EELV data measured using the FRC INview module of the ventilator. The calculation of PEEP_bolus,equiv_ and ΔEELV_bolus,equiv_ is described in detail in the Supplementary Information.

The values are presented as median and interquartile range (Q_1_-Q_3_) unless stated otherwise. The differences in regional ventilation, EELV, EVLW and ITBV were compared using Friedman’s test with Tukey’s post-hoc analysis. Wilcoxon’s test was used for the comparison of the differences between the measured and the estimated EELV values, and for the comparison of initial and end values of ventilatory parameters. A *p*-value < 0.05 was considered statistically significant.

## Results

**Figure 3:**
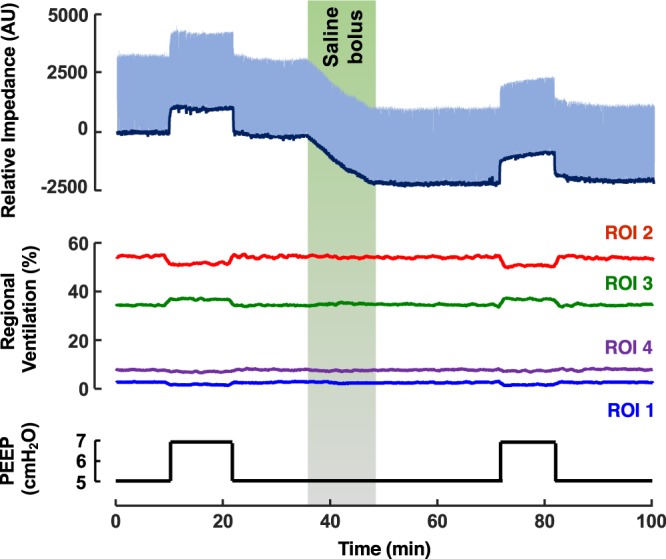
Typical changes of end-expiratory lung impedance and regional ventilation during the animal trial. Top graph: End-expiratory lung impedance (EELI) trend (dark blue) was determined as local minima of the relative impedance waveform (light blue). Middle graph: distribution of ventilation in the pre-defined regions of interest (ROIs). Bottom graph: time course of positive end-expiratory pressure (PEEP) during the trial.

All animals completed the full study protocol and were included in the data analysis. Decrease in end-expiratory lung impedance (EELI) was observed in all subjects when the bolus of normal saline was administered intravenously. A representative example of changes in EELI, the time course of regional ventilation in pre-defined ROIs and the corresponding values of applied PEEP are shown in Fig. 3.

**Figure 4:**
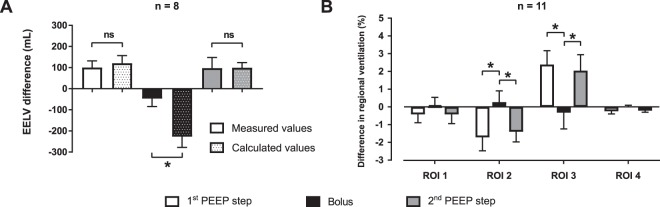
Changes in end-expiratory lung volume (EELV) and regional ventilation. (**A**) Ventilator-measured changes of EELV during the bolus of normal saline and induced by the PEEP steps together with the estimated EELV changes calculated from the EIT data. The solid black bar represents the decrease of EELV during the administration of normal saline (ΔEELV_bolus,meas_). The dotted black bar represents the respective change of EELV estimated from the EIT data (ΔEELV_bolus,equiv_). (**B**) Differences in regional ventilation caused by the PEEP steps and by the bolus of normal saline. Statistically significant differences (Wilcoxon’s test for the EELV data, Friedman’s test with Tukey’s post hoc analysis for the regional ventilation data, *p* < 0.05) are denoted with *. The error bars represent standard deviation.

The impedance change caused by the administration of the normal saline (ΔZ_bolus_) was equivalent to a decrease of end-expiratory lung volume (ΔEELV_bolus,equiv_) by 227 mL (178–250 mL). Compared to this, the measured decrease of end-expiratory lung volume (ΔEELV_bolus,meas_) was only 52 mL (25–78 mL) and differed significantly from the values of ΔEELV_bolus,equiv_, as depicted in Fig. 4A. When expressed as a value of an equivalent (virtual) positive end-expiratory pressure (PEEP_bolus,equiv_), the administration of normal saline was equivalent to the decrease of PEEP by 4.40 cmH_2_O (3.95–4.59 cmH_2_O).

**Figure 5:**
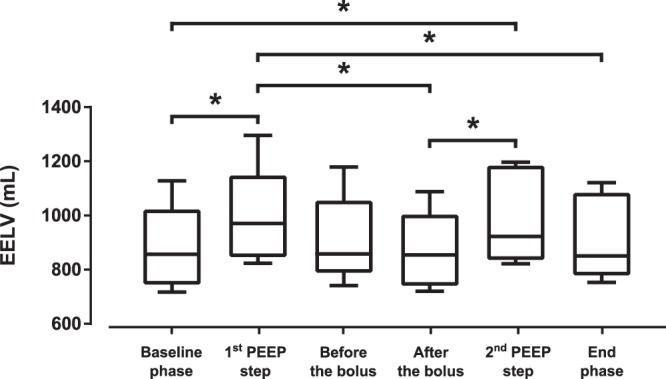
End-expiratory lung volume (EELV) measured by FRC INview module of the ventilator during the experiment. Statistically significant differences (Friedman’s test with Tukey’s post hoc analysis, *p* < 0.05) are denoted with *.

**Figure 6:**
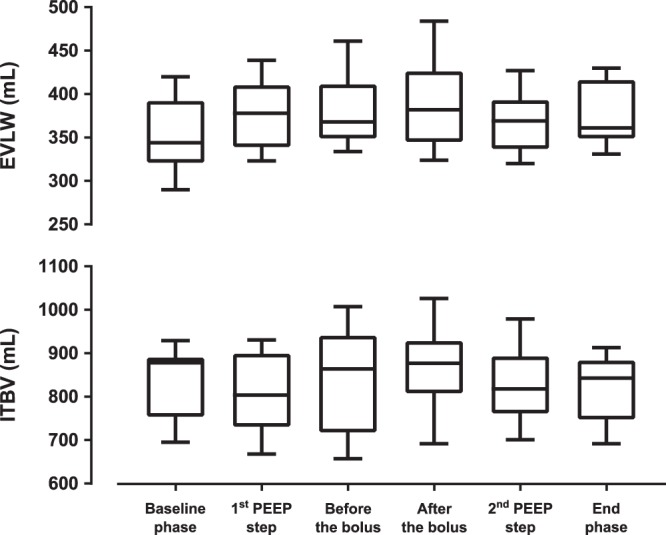
Extravascular lung water (EVLW) and intrathoracic blood volume (ITBV) during the experiment. No statistically significant differences were observed in the values of EVLW and ITBV (Friedman’s test, *p* > 0.05).

The administration of saline bolus was not associated with any statistically significant difference in the regional distribution of lung ventilation and in EELV, as shown in Figs 4 and 5. Contrary to this, the PEEP steps performed during the study protocol induced significant differences in the regional ventilation in ROI 2 and 3, as shown in Fig. 4B and in EELV, as shown in Fig. 5. The ventilatory parameters remained stable during the experiment, except for peak airway pressure and compliance in which statistically insignificant changes were observed (Table 1). No statistically significant differences were observed in the values of EVLW and ITBV measured by TPTD during the experiment, as shown in Fig. 6.

## Discussion

The main finding of this study is that fast administration of normal saline significantly affects end-expiratory lung impedance (EELI) provided by EIT and thus can compromise assessment of end-expiratory lung volume. The study also shows that the administration of therapeutic volume of normal saline causes a decrease in EELI comparable to a decrease of PEEP by several cmH_2_O. Nevertheless, the administration of normal saline does not affect regional distribution of lung ventilation in the pre-defined regions of interest.

Statistically significant shift of regional ventilation towards dorsal parts of lungs (increased proportional ventilation in ROI 3 and its decrease in ROI 2) was observed after the PEEP was increased by 2 cmH_2_O. Compared to that, the application of normal saline was not associated with remarkable changes in the regional distribution of lung ventilation determined by EIT. This finding indicates that there was no redistribution of lung ventilation or regional differences of lung impedance when the saline was administered intravenously. PEEP-induced increase of EELV led to an increase of regional lung ventilation in dependent parts of the lungs which is the main mechanism why PEEP optimisation represents a basic strategy for homogenisation of lung ventilation. Therefore, the EELI changes caused by the alterations of EELV can be distinguished from the changes of EELI caused by the administration of normal saline by analysing the regional differences in ventilation between dependent and non-dependent lung regions (ROIs).

Statistically significant differences in EELV were observed only when PEEP was modified. The application of normal saline did not result in statistically significant change of EELV when measured by the ventilator. Compared to that, the change of EELV estimated from the decrease of EELI differed significantly also during the application of the bolus of normal saline. This finding implies that administration of normal saline might be potentially misinterpreted as derecruitment, especially when regional ventilation is not taken into account.

A slight decrease of compliance and an increase of peak airway pressure were observed during the study. Though not statistically nor clinically significant, the authors speculate that these changes might be caused by a slight increase of the volume of intrathoracic water. This finding correlates with observed insignificant increase of EVLW. Moreover, gradual increase in the portion of atelectatic lung regions can be assumed at the end phase of the experiment due to the mechanical lung ventilation, muscle relaxation and anaesthesia^16^. Considering the magnitude of the observed changes in ventilatory parameters, the described finding may be also affected by the imprecision of the ventilator measurements.

No significant changes were observed in the values of EVLW and ITBV determined by transpulmonary thermodilution. The decrease of EELI observed during the administration of normal saline thus can be explained by an increase of the compartment with higher electrical conductivity. The conductivity of lung tissue may be affected either by the volume of the administered fluid or by its electric properties. It has been described that hypertonic saline solution can be used as a contrast agent for EIT^17^. Our findings demonstrate that, compared to TPTD, EIT is more sensitive to identify changes in intrathoracic fluids when normal saline is administered.

The administered volumes of crystalloids per unit of body weight were considerably lower when compared to the volumes that are used for fluid therapy in clinical practice. For example, we used 10.64 mL/kg (9.90–10.99 mL/kg), while the guidelines of Surviving Sepsis Campaign recommend the fluid challenge of at least 30 mL/kg of crystalloids for the first three hours of the initial phase of the sepsis or septic shock treatment^18^. However, the mean dosing rate applied in our study (103 mL/kg/h) was substantially higher than the dosing rate recommended by the guidelines (>30 mL/kg within the first 3 hours, i.e. >10 mL/kg/h). Our main reason for the choice of the fast dosing rate and a short dosing time was to separate the effect of the saline administration from possible derecruitment, interferences or time instability of EIT^19^.

When studying the response time of the bolus administration upon the trend of end-expiratory lung impedance, we did not observe any remarkable time delay between the beginning of the saline administration and the onset of impedance change ΔZ_bolus_.

The PEEP step of 2 cmH_2_O was used because it is a common step used for PEEP titration^20,21^. The aim of the performed PEEP steps in our protocol was to obtain changes of EELI that can be further used for conversion of ΔZ_bolus_ into the values of equivalent (virtual) PEEP step (PEEP_bolus,equiv_). Large PEEP changes were avoided due to possible alterations of lung mechanics. Moreover, applying a small PEEP step allowed us to linearize the relationship between PEEP and EELI, which in general is non-linear^22^. The duration of PEEP steps was based on clinical studies that investigated the effect of PEEP on changes of EELV by means of EIT^23,24^. Longer duration of the PEEP steps was not necessary because the levels of EELI became stable after several breath cycles.

There are several studies that investigated the effect of fluid-related interventions upon the changes in thoracic impedance^12–15^. However, none of these studies compared the magnitude of the fluid-related impedance changes with the impedance changes caused by lung ventilation. Our study compares the impedance changes caused by administration of normal saline with those that are associated with alterations in end-expiratory lung volume. These changes were assessed quantitatively and were described in time domain as well as on a regional basis.

The results indicate that, compared to PEEP alterations, the administration of normal saline bolus does not affect regional distribution of lung ventilation when assessed by EIT. This finding is in agreement with the study of Bodenstein *et al*.^15^ who investigated the effect of volume interventions on pulmonary bioimpedance. The authors demonstrated that neither infusion of crystalloid or colloid, nor blood withdrawal cause changes in regional lung ventilation. However, changes in regional lung ventilation can be used for determination of extravascular lung water when small rotations of the subject are applied, as showed by Trepte *et al*.^25^. Trepte *et al*. detected changes in tidal variations between left and right hemithorax in animals with induced lung injury when gravity-dependent redistribution of pulmonary oedema was created by lateral rotation of the animals along their longitudinal axis.

Using healthy animals as the subjects of the study, we assumed that the portion of ventilated lung was within the physiological limit and remained stable in the phases with constant PEEP values. To align the study closer to the real needs of clinical practice it would be appropriate to conduct the study in pathophysiological lung conditions such as acute respiratory distress syndrome^26,27^. The effect of such conditions is not easy to predict. On one hand, increased permeability of the alveolar-capillary barrier could further induce extravasation of water in the interstitial and alveolar space with more pronounced and stable decrease of EELV and EELI and an increase of EVLW. On the other hand, the amount of intra-thoracic water might have already been increased during ARDS and thus the additional reduction of thoracic impedance caused by the fluid bolus may be minimised with a little or no change in EELI.

Seen from clinical perspective, the evaluation of lung recruitment and derecruitment that is based only on the information from EELI trends may be misleading when fluids are being administered intravenously. Therefore, assessment of changes in regional distribution of lung ventilation should be prioritized in such situations, since no effect of fluid administration on regional ventilation was observed in our study.

### Limitations

The application of only one fluid type, its volume and infusion rate is a certain limitation of the study. To assure reliable quantification of the studied effect, we prioritized using one specific setting to achieve sufficient statistical power rather than investigating wider range of conditions, potentially resulting in underpowered study. Our previous study showed a linear decrease of EELI when either blood or Ringer’s solution was administered intravenously^28^. Such finding indicates that the administration of various fluid volumes would result in a linearly proportional decrease of EELI. Similarly, one may assume that application of different dosing rates would lead to the proportional change in the steepness of the EELI decrease. To fully explore the effect of fluid type, volume, infusion rate and different PEEP levels, more extensive randomized interventional trial would be necessary.

## Conclusion

Fast intravenous administration of normal saline bolus causes significant changes in the values of end-expiratory lung impedance provided by electrical impedance tomography. The impedance changes induced by the bolus administration are comparable to the changes of EELV that would be caused by a decrease of PEEP in the order of several units of cmH_2_O. The fast administration of the saline bolus has no effect on the distribution of regional lung ventilation. Seen from clinical perspective, the assessment of EELV changes that is based only on the information from EELI trend may be misleading when normal saline is administered intravenously.

## Supplementary information

Supplementary information

## Data Availability

The data generated and analysed during the current study will be made available from the corresponding author on reasonable request.

## References

[1] Frerichs I (2017). Chest electrical impedance tomography examination, data analysis, terminology, clinical use and recommendations: consensus statement of the Translational EIT development study group. Thorax..

[2] Wrigge H (2008). Electrical impedance tomography compared with thoracic computed tomography during a slow inflation maneuver in experimental models of lung injury. Crit. Care Med..

[3] Wolf GK (2012). Reversal of dependent lung collapse predicts response to lung recruitment in children with early acute lung injury. Pediatr. Crit. Care Med..

[4] Muders T (2012). Tidal recruitment assessed by electrical impedance tomography and computed tomography in a porcine model of lung injury. Crit. Care Med..

[5] Odenstedt H (2005). Slow moderate pressure recruitment maneuver minimizes negative circulatory and lung mechanic side effects: evaluation of recruitment maneuvers using electric impedance tomography. Intensive Care Med..

[6] Lowhagen K, Lindgren S, Odenstedt H, Stenqvist O, Lundin S (2011). Prolonged moderate pressure recruitment manoeuvre results in lower optimal positive end-expiratory pressure and plateau pressure. Acta Anaesthesiol. Scand..

[7] Erlandsson K, Odenstedt H, Lundin S, Stenqvist O (2006). Positive end-expiratory pressure optimization using electric impedance tomography in morbidly obese patients during laparoscopic gastric bypass surgery. Acta Anaesthesiol. Scand..

[8] Becher TH (2014). Assessment of respiratory system compliance with electrical impedance tomography using a positive end-expiratory pressure wave maneuver during pressure support ventilation: a pilot clinical study. Crit. Care..

[9] Blankman P, Hasan D, Erik GJ, Gommers D (2014). Detection of the ‘best’ positive end-expiratory pressure derived from electrical impedance tomography parameters during a decremental positive end-expiratory pressure trial. Crit. Care..

[10] Kushner RF, Schoeller DA (1986). Estimation of total body water by bioelectrical impedance analysis. Am. Journal Clin. Nutr..

[11] Tang W, Ridout D, Modi N (1997). Assessment of total body water using bioelectrical impedance analysis in neonates receiving intensive care. Arch. Dis. Child Fetal Neonatal. Ed..

[12] Sadleir RJ, Fox RA (2001). Detection and quantification of intraperitoneal fluid using electrical impedance tomography. IEEE Trans. Biomed. Eng..

[13] Tucker AS, Ross EA, Paugh-Miller J, Sadleir RJ (2011). *In vivo* quantification of accumulating abdominal fluid using an electrical impedance tomography hemiarray. Physiol. Meas..

[14] Noble TJ, Harris ND, Morice AH, Milnes P, Brown BH (2000). Diuretic induced change in lung water assessed by electrical impedance tomography. Physiol. Meas..

[15] Bodenstein M (2012). Influence of crystalloid and colloid fluid infusion and blood withdrawal on pulmonary bioimpedance in an animal model of mechanical ventilation. Physiol Meas..

[16] Laghi, F. & Tobin, M. J. Indications for mechanical ventilation in *Principles and practice of mechanical ventilation* (ed. Tobin, M. J.) 115 (The McGraw-Hill Companies, Inc., 2013).

[17] Frerichs I (2002). Regional lung perfusion as determined by electrical impedance tomography in comparison with electron beam CT Imaging. IEEE Trans. Med. Imaging..

[18] Rhodes A (2017). Surviving sepsis campaign: international guidelines for management of severe sepsis and septic shock: 2016. Intensive Care Med..

[19] Frerichs I (2011). Patient examinations using electrical impedance tomography – sources of interference in the intensive care unit. Physiol. Meas..

[20] Costa ELV (2009). Bedside estimation of recruitable alveolar collapse and hyperdistension by electrical impedance tomography. Intensive Care Med..

[21] Karsten J, Grusnick C, Paarmann H, Heringlake M, Heinze H (2015). Positive end-expiratory pressure titration at bedside using electrical impedance tomography in post-operative cardiac surgery patients. Acta Anaesthesiol. Scand..

[22] Bikker IG, Leonhardt S, Bakker J, Gommers D (2009). Lung volume calculated from electrical impedance tomography in ICU patients at different PEEP levels. Intensive Care Med..

[23] Grivans C, Lundin S, Stenquist O, Lindgren S (2011). Positive end-expiratory pressure-induced changes in end-expiratory lung volume measured by spirometry and electric impedance tomography. Acta Anaesthesiol. Scand..

[24] Markhorst DG, Groeneveld AB, Heethaar RM, Zonneveld E, van Genderingen HR (2009). Assessing effects of PEEP and global expiratory lung volume on regional electrical impedance tomography. J. Med. Eng. Technol..

[25] Trepte CJC (2016). Electrical impedance tomography (EIT) for quantification of pulmonary edema in acute lung injury. Crit. Care..

[26] Otáhal M, Mlček M, Vítková I, Kittnar O (2016). A novel experimental model of acute respiratory distress syndrome in pig. Physiol. Res..

[27] Pomprapa A (2014). Automatic protective ventilation using the ARDSNet protocol with the additional monitoring of electrical impedance tomography. Crit. Care..

[28] Suchomel J, Sobota V (2013). A model of end-expiratory lung impedance dependency on total extracellular body water. J. Phys.: Conf. Ser..

