# Supplementary material for: Intravenous administration of normal saline may be misinterpreted as a change of end-expiratory lung volume when using electrical impedance tomography
Source: Sci Rep. 2019 Apr 8;9:5775. doi: 10.1038/s41598-019-42241-7 (PMC6453964; doi:10.1038/s41598-019-42241-7)
Supplement: Supplementary file 1 — Supplementary information [file 41598_2019_42241_MOESM1_ESM.pdf]

Intravenous administration of normal saline may be misinterpreted as a change of end-expiratory lung volume when using electrical impedance tomography

Vladimír Sobota[1,2], Martin Müller[1,3], Karel Roubík[1]

[1]Department of Biomedical Technology, Faculty of Biomedical Engineering, Czech Technical University in Prague, Kladno, Czech Republic.

[2]Department of Physiology, Maastricht University, Maastricht, The Netherlands.

[3] Department of Anaesthesiology and Intensive Care, First Faculty of Medicine, Charles University and Thomayer Hospital, Prague, Czech Republic.

Anaesthesia induction

The animals were premedicated with azaperone (2 mg/kg IM), or alternatively with midazolam (0.1 to 0.2 mg/kg IM) and atropine sulphate (0.05 to 0.1 mg/kg IM). Anaesthesia was initiated with ketamine hydrochloride (20 mg/kg IM), followed by boluses of morphine (0.1 mg/kg IV) and propofol (2 mg/kg IV). After the anaesthesia induction, animals were orotracheally intubated by a cuffed endotracheal tube (inner diameter 7.5 mm) and connected to the conventional ventilator (Engström Carestation, GE Healthcare, Waukesha, WI, USA) in the supine position. Anaesthesia was maintained with propofol (8 to 10 mg/kg/h IV) in combination with morphine (0.1 mg/kg/h IV). To suppress spontaneous breathing, muscle relaxant pipecuronium bromide (4 mg boluses every 45 min IV) was administered.

Differences in regional ventilation

For each breath cycle, tidal variation (TV) image was calculated as a difference between the end-inspiratory and the previous end-expiratory EIT image. Four horizontal regions of interest (ROIs) were determined in the TV images, numbered from 1 (ventral) to 4 (dorsal). Proportional ventilation was calculated for each ROI and was expressed as a percentage of the overall tidal impedance change. Mean values of proportional ventilation were calculated for 6 different intervals, each in a duration of 60 seconds as shown in Figure E1. The differences in regional ventilation were calculated for both PEEP steps and the bolus:

- 1st PEEP step:

$$\mathrm{ROI\#}_{\mathrm{PEEP,\ before}} = \mathrm{ROI\#}_2 - (\mathrm{ROI\#}_1 + \mathrm{ROI\#}_3)/2$$

- Saline bolus:

$$\mathrm{ROI\#}_{\mathrm{bolus}} = \mathrm{ROI\#}_3 - \mathrm{ROI\#}_4$$

- 2nd PEEP step:

$$\mathrm{ROI\#}_{\mathrm{PEEP,\ after}} = \mathrm{ROI\#}_5 - (\mathrm{ROI\#}_4 + \mathrm{ROI\#}_6)/2$$

The symbol # indicates the region of interest number (1-4) and the lower index represents the interval depicted in Figure E1. The resulting differences in regional ventilation are shown in Figure 4B in the manuscript.

Calculation of the change of end-expiratory lung volume equivalent to the administration of saline bolus ($\Delta EELV_{bolus,equiv}$)

The change of end-expiratory lung impedance ΔZ_{bolus} was expressed as an equivalent change of end-expiratory lung volume $\Delta EELV_{bolus,equiv}$. For each subject, mean tidal variations $\Delta Z_{TV,j}$ were determined from six pre-defined intervals in the EIT record, as shown in Figures E1 and E2. Each interval had a duration of 60 seconds. Using the corresponding mean tidal volumes $V_{T,j}$, the value of $\Delta EELV_{bolus,equiv}$ was calculated as follows:

$$\Delta \mathrm{EELV}_{\mathrm{bolus,equiv}} = -\Delta \mathrm{Z}_{\mathrm{bolus}} \cdot \frac{1}{6} \sum_{j=1}^{6} \frac{\mathrm{V}_{T,j}}{\Delta \mathrm{Z}_{\mathrm{TV},j}}.$$

The resulting value of $\Delta EELV_{bolus,equiv}$ is shown in the Figure 4A in the manuscript (dotted black bar).

Calculation of the end-expiratory lung volume changes caused by the PEEP steps

The mean changes of end-expiratory lung impedance caused by the 1st PEEP step ($\Delta Z_{1stPEEP}$) and by the 2nd PEEP step ($\Delta Z_{2ndPEEP}$) were calculated as follows:

$$\Delta \mathrm{Z}_{\mathrm{1stPEEP}} = \frac{1}{2} (\Delta \mathrm{Z}_{\mathrm{PEEP,1}} + \Delta \mathrm{Z}_{\mathrm{PEEP,2}}),$$

$$\Delta \mathrm{Z}_{\mathrm{2ndPEEP}} = \frac{1}{2} (\Delta \mathrm{Z}_{\mathrm{PEEP,3}} + \Delta \mathrm{Z}_{\mathrm{PEEP,3}}).$$

The definition of the impedance changes $\Delta Z_{PEEP,1\text{-}4}$ is depicted in the Figure E2. The equivalent changes of end-expiratory lung volume caused by the 1st and the 2nd PEEP step were calculated as follows:

$$\Delta\mathrm{EELV}_{\mathrm{1stPEEP,equiv}} = \Delta\mathrm{Z}_{\mathrm{1stPEEP}} \cdot \frac{1}{6}\sum_{j=1}^{6} \frac{\mathrm{V}_{T,j}}{\Delta\mathrm{Z}_{\mathrm{TV},j}},$$

$$\Delta\mathrm{EELV}_{\mathrm{2ndPEEP,equiv}} = \Delta\mathrm{Z}_{\mathrm{2ndPEEP}} \cdot \frac{1}{6}\sum_{j=1}^{6} \frac{\mathrm{V}_{T,j}}{\Delta\mathrm{Z}_{\mathrm{TV},j}}.$$

The resulting values of $\Delta EELV_{1stPEEP,equiv}$ and $\Delta EELV_{2ndPEEP,equiv}$ are shown in the Figure 4A in the manuscript (the dotted white bar represents $\Delta EELV_{1stPEEP,equiv}$, the dotted grey bar represents $\Delta EELV_{2ndPEEP,equiv}$).

Calculation of the virtual PEEP step equivalent to the administration of normal saline bolus ($PEEP_{bolus,equiv}$)

For each subject, the impedance change ΔZ_{bolus} caused by the administration of 500 mL of normal saline was expressed as a value of equivalent virtual PEEP step (denoted as $PEEP_{bolus,equiv}$). The values of impedance changes $\Delta Z_{PEEP,1\text{-}4}$ caused by the respective PEEP changes $\Delta PEEP_{1\text{-}4}$ were determined as shown in Figure E2. The value of $PEEP_{bolus}$ was then obtained as follows:

$$\mathrm{PEEP}_{\mathrm{bolus,equiv}} = \Delta\mathrm{Z}_{\mathrm{bolus}} \cdot \frac{1}{4}\sum_{i=1}^{4} \left|\frac{\Delta\mathrm{PEEP}_{i}}{\Delta\mathrm{Z}_{\mathrm{PEEP},i}}\right|.$$

The expression of ΔZ_{bolus} as both $PEEP_{bolus}$ and $\Delta EELV_{bolus}$ allows to compare the magnitudes of relative impedance changes caused by the PEEP maneuvers with the effect of normal saline administration.

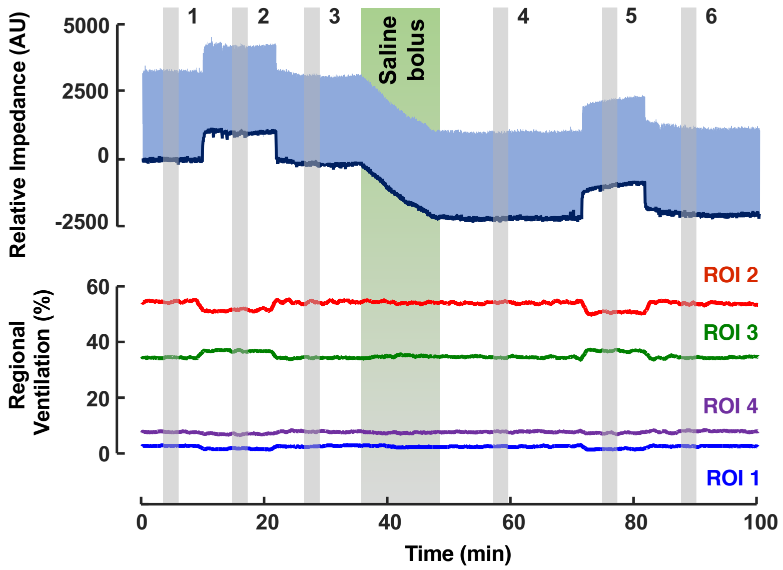

Figure E1 Definition of six intervals for calculation of differences in regional ventilation and equivalent change of end-expiratory lung volume ($\Delta EELV_{bolus,equiv}$). Top graph: a representative relative impedance waveform. Bottom graph: distribution of ventilation in the pre-defined regions of interest (ROIs). The intervals are depicted in grey.

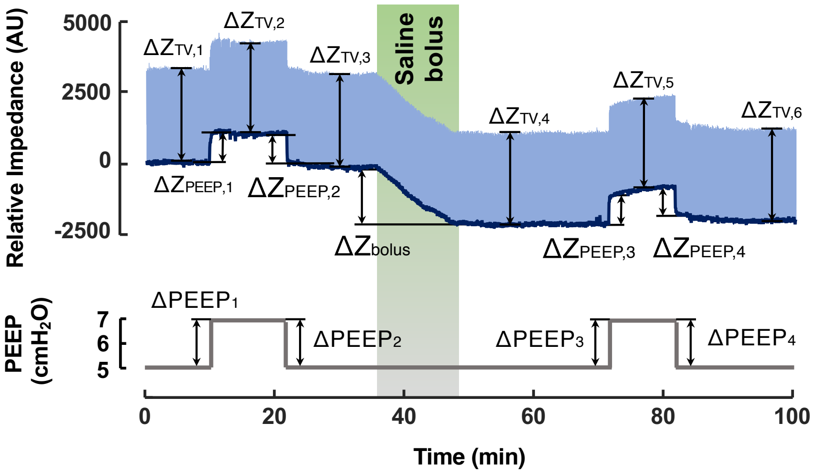

Figure E2 Definition of variables for calculation of equivalent change in end-expiratory lung volume ($\Delta EELV_{bolus,equiv}$) and virtual positive end-expiratory pressure step ($PEEP_{bolus,equiv}$). Top graph: a representative relative impedance waveform with depicted end-expiratory lung impedance trend (dark blue). Bottom graph: a time course of positive end-expiratory pressure (PEEP).
